# Children’s rare disease cohorts: an integrative research and clinical genomics initiative

**DOI:** 10.1038/s41525-020-0137-0

**Published:** 2020-07-06

**Authors:** Shira Rockowitz, Nicholas LeCompte, Mary Carmack, Andrew Quitadamo, Lily Wang, Meredith Park, Devon Knight, Emma Sexton, Lacey Smith, Beth Sheidley, Michael Field, Ingrid A. Holm, Catherine A. Brownstein, Pankaj B. Agrawal, Susan Kornetsky, Annapurna Poduri, Scott B. Snapper, Alan H. Beggs, Timothy W. Yu, David A. Williams, Piotr Sliz

**Affiliations:** 1 Computational Health Informatics Program, Boston Children’s Hospital, Boston, MA 02115 USA; 2 The Manton Center for Orphan Disease Research, Boston Children’s Hospital, Boston, MA 02115 USA; 3 Harvard Medical School, Boston, MA 02115 USA; 4 Department of Neurology, F.M. Kirby Neurobiology Center, Boston Children’s Hospital, Boston, MA 02115 USA; 5 Division of Epilepsy and Clinical Neurophysiology and Epilepsy Genetics Program, Boston Children’s Hospital, Boston, MA 02115 USA; 6 Division of Gastroenterology, Hepatology and Nutrition, Boston Children’s Hospital, Boston, MA 02115 USA; 7 Division of Genetics and Genomics, Boston Children’s Hospital, Boston, MA 02115 USA; 8 Division of Newborn Medicine, Boston Children’s Hospital, Boston, MA 02115 USA; 9 Research Administration, Boston Children’s Hospital, Boston, MA 02115 USA; 10 Division of Hematology/Oncology, Dana-Farber/Boston Children’s Cancer and Blood Disorders Center, Boston, MA 02115 USA

**Keywords:** Personalized medicine, Genetic databases, Medical genomics, Paediatrics, Data processing

## Abstract

While genomic data is frequently collected under distinct research protocols and disparate clinical and research regimes, there is a benefit in streamlining sequencing strategies to create harmonized databases, particularly in the area of pediatric rare disease. Research hospitals seeking to implement unified genomics workflows for research and clinical practice face numerous challenges, as they need to address the unique requirements and goals of the distinct environments and many stakeholders, including clinicians, researchers and sequencing providers. Here, we present outcomes of the first phase of the Children’s Rare Disease Cohorts initiative (CRDC) that was completed at Boston Children’s Hospital (BCH). We have developed a broadly sharable database of 2441 exomes from 15 pediatric rare disease cohorts, with major contributions from early onset epilepsy and early onset inflammatory bowel disease. All sequencing data is integrated and combined with phenotypic and research data in a genomics learning system (GLS). Phenotypes were both manually annotated and pulled automatically from patient medical records. Deployment of a genomically-ordered relational database allowed us to provide a modular and robust platform for centralized storage and analysis of research and clinical data, currently totaling 8516 exomes and 112 genomes. The GLS integrates analytical systems, including machine learning algorithms for automated variant classification and prioritization, as well as phenotype extraction via natural language processing (NLP) of clinical notes. This GLS is extensible to additional analytic systems and growing research and clinical collections of genomic and other types of data.

## Introduction

Ten million children are affected by a rare disease with a genetic etiology^1,2^. Yet, treatments for rare diseases are scarce due to the unique challenges of rare disease research^1,3^, including the small size of patient cohorts and the identification of precise genetic etiologies of disease. Of the demonstrated applications of genomics for patient care in rare disease cohorts^4–8^, most do not address the specific challenges of the pediatric population. Rare disease pediatric patients are few in number and tend to be geographically widely distributed^9^, and therefore combining small cohorts within an institution or with other institutions can be valuable^7,8,10–15^. Existing inter-institutional networks have increasingly focused on integration of genomic data, with some focused on pediatric populations^16,17^ and others focused on national enrollment^12,13^. Therefore it is increasingly important for individual institutions to develop approaches that are compatible with integration to these larger efforts.

Institutional and national initiatives face common challenges in their decisions around patient selection, sequencing and analyses. These decisions shape gene discovery and genetic diagnosis outcomes. Family-based or trio-based sequencing has been shown to be substantially more effective in various patient populations than singleton sequencing,^18–24^ and researchers can identify rare variation leveraging extended pedigrees^25,26^. Whole genome sequencing (WGS) and whole exome sequencing (WES) have been shown to be more effective at diagnosing patients with suspected genetic diseases than other technologies^20,27–30^. Hospital-based sequencing analyses have been shown to be more effective than reference-lab-based analyses,^27^ because it often occurs at academic medical centers and in a research environment where analysts have more access to deep phenotypic information about patients, better ability to re-contact patients and their clinicians, and more domain-specific expertise. Many studies have also argued in support of the added utility of iterative re-analyses of sequencing data^31–38^ and highlighted the importance of these factors in^20,27,28^ pediatric and neonatal populations^18–20,36,38–41^. Institutional and national initiatives must consider additional questions around consenting mechanisms and language. Initiatives may opt to sequence patients using research sequencing or Clinical Laboratory Improvement Amendments (CLIA)-compliant standards. The computational burden of analyzing genomic data raises questions regarding how to develop infrastructure to analyze data.

Of all of the challenges facing the community, perhaps none is as profound as that of consenting, particularly in the realm of data provenance and use. Within institutions, and even more so as data sources are combined across institutions, incompatibilities in research consenting practices limit the ability to combine data sources, especially given that once consent is obtained it cannot be readily modified. Many consent forms developed in the past and still used today do not support broad use of research data and instead restrict data use to disease-specific research, creating barriers to sharing data within and between institutions. Many studies have shown that most research participants are supportive of data sharing across the research community^42–47^. Data sharing issues are widely recognized and have prompted efforts such as Platform for Engaging Everyone Responsibly (PEER),^48^ National Institutes of Health’s Genomic Data Sharing Policy,^49^ and the Global Alliance for Genomics and Health (GA4GH) Data Use Ontology^50^ to find more elegant solutions. Adopting those recommendations across an institution (including modifying existing studies) would accelerate general readiness to engage in academic collaborations within the United States and internationally. During this adoption process, there is also additional opportunity to align consenting with the specific needs of clinical processes including access to identified data, clinical confirmation of findings, and access to data by patients.

Although hospital-based analyses frequently leverage genomic data together with phenotypic information collected through clinical practice and by the research laboratory, these data typically remain siloed in multiple locations^51^. While valid privacy and regulatory policy considerations have historically necessitated the separation of data from the laboratory and the clinic, as well as data from different cohorts, these distinctions pose barriers to the utilization of multiple data sources to accelerate scientific discoveries and pediatric precision medicine. Whole exome and genome sequencing data are not yet readily incorporated into patient medical records^52,53^, and even the limited variant data contained in clinical reports from reference laboratories are often stored by Electronic Health Records (EHRs) in a manner that is not automatically searchable (e.g., as scanned PDFs). Thus, though re-analyses, and in particular hospital-based re-analyses, of clinical sequencing data can lead to improved gene discovery and diagnosis rates, these re-analyses rarely happen in practice. These realities necessitate the creation of institutional data warehouses to store genomic and phenotypic information and provide unified infrastructure for the interrogation of full raw sequencing data, which is otherwise impossible or very time consuming for researchers and clinicians. Increasingly, research and clinical genomics overlap to provide patients with the latest discoveries, particularly in the translation of research findings to the clinic and in the treatment of raw sequencing data like other unprocessed measurements, reviewable by clinicians. As research and clinical sequencing require similar infrastructure to review genomic data, there is an opportunity to foster intra-institutional synergy through use of a unified platform that accommodates the unique regulatory requirements of each.

Patient enrollment in genomic research has been ongoing at BCH for close to three decades, yet data generated across research teams have historically not been available for collective analysis. Here, we describe the Children’s Rare Disease Cohorts (CRDC) initiative, a program at BCH integrating genomic, research, and clinical data to expedite pediatric precision medicine. Within its first year, the CRDC launched 15 studies of rare pediatric-onset Mendelian diseases, including epilepsy and inflammatory bowel disease (IBD). Together, these studies enrolled 2441 participants and collected genotypic data and phenotypic data from the medical record and research surveys. We developed a genomics learning system (GLS) for storage and use of the data. By developing institutional infrastructure, leveraging a uniform sequencing provider, and aligning IRB protocols with consistent and broad consenting principles, the de-identified genomic and phenotypic data of the CRDC was made available for collective analysis across the institution in the GLS. In addition to supporting the CRDC, the GLS was used to analyze clinical and research data of an additional set of 6075 exomes and 112 genomes. CRDC data in the GLS are accessible to translational researchers for gene discovery, to basic science researchers for functional studies of candidate genes, and to clinicians for accelerated hospital-based patient diagnoses.

## Results

### Process

The mechanisms, processes and technologies implemented at BCH during the first phase of the CRDC were focused on enabling hospital-based analysis and re-analysis of genomic data to accelerate rates of discovery and patient diagnoses. This project involved a strategic, internally-funded investment in CLIA-compliant research sequencing of disease-specific cohorts. The project was informed by a series of faculty town hall meetings and the formation of a Genomics Blue Ribbon Committee who developed specific recommendations to the hospital leadership that resulted in institutional funding. Subsequently, a cohorts sub-committee was formed, which surveyed the BCH community in October 2017 requesting estimates regarding cohorts of pediatric patients that would benefit from sequencing. A total of 26,148 patients in 83 disease cohorts from 23 departments or divisions were estimated with an additional 4692 patients predicted to be diagnosed with those diseases at BCH in 2018. Two of these cohorts (epilepsy and IBD) anticipated high diagnosis rates (500 patients each) in 2018 and were selected by the cohort committee to pilot processes and technologies for cohort sequencing. Sample collection started in October 2018 and by January 2019, the cohort committee initiated a request for application (RFA) for additional cohorts. Applications from investigators across the institution included 36 investigators in 19 departments regarding 11,810 patients with 7987 that would be eligible for enrollment in 2019. An additional 13 pediatric-onset Mendelian disease cohorts were selected from the applications, expanding the engagement of the CRDC in phase I (October 2018-September 2019) to 15 cohorts. Implementation of phase I of CRDC was completed as a multidisciplinary effort across research teams, institutional leadership, the Research Computing (RC) group, which is embedded within the Information Services Department and affiliated with the Computational Health Informatics Program (CHIP), and members of the Manton Center for Orphan Disease Research^54^.

### Consenting alignment

The integration of data sources at scale for collective analysis necessitated the development of standard approaches to consenting patients and family members. In order to facilitate data sharing at BCH, the CRDC implementation team developed a consenting framework that aligned with the GA4GH guidelines for international data sharing^55^. This consenting framework was subsequently integrated into 15 IRB protocols and informed consent documents. The framework was designed to address four major aspects: (1) to empower the rights and interests of the research participants, (2) to support sample and data flow considerations, (3) to enable the use of data across the institution, and (4) to facilitate engagement with other academic networks and industry sponsors to accelerate discovery and therapeutics development (Table 1 and Supplementary Table 1). Many of these aspects were already partially incorporated into the first two participating research teams’ protocols, as well as other research protocols at BCH, such as the Manton Center and the Precision Link Biobank. Modifications of the first two protocols streamlined facets of CLIA-compliant sequencing and data return processes. The framework was comprised of informed consent, protocol, and IRB form language templates that could be added to existing research protocols or incorporated into new protocols. All participants were consented to be re-contacted to request additional data/samples and regarding their interest in being offered enrollment in other research studies. In order to support potential clinical follow-up, patients were offered the option to list a clinician in the research consent form to be involved in potential clinical orders. The framework was reviewed by the BCH IRB in the context of the first two protocols, and approved with the understanding that it would be suitable for other protocols. As a result of the RFA, the framework was applied to 13 other protocols, many of which were originally not in alignment with the framework, and approved by the IRB (Supplementary Table 2).

**Table 1:** Summary of protocol changes implemented by the CRDC to support patient rights and operations.

Aspects	Consenting principles implemented under CRDC	Use in consent forms prior to CRDC on-boarding
Rights and interests of the patient	Identified variants are clinically confirmed	Sometimes
Rights and interests of the patient	Patient opts for return of primary results, primary and secondary/incidental, or neither	Very rarely
Rights and interests of the patient	Patient consents to re-contact to request additional data/samples and being offered enrollment in other research studies	Sometimes
Rights and interests of the patient	Patient data protected by NIH certificate of confidentiality	Rarely
Sample and data flow	Research consents contain language regarding the use of previously collected clinical data	Very rarely
Sample and data flow	Remote consenting and e-consenting are available	Very rarely
Sample and data flow	Supports Biobanking at the BCH Biobank	Rarely
Sample and data flow	Consent allows the identification of genetic factors	Sometimes
Sample and data flow	Consent enables identified CLIA sequencing upfront for streamlined confirmation	Never
BCH data use (secondary use, control sample across population)	Samples and data (genomic sequences, medical record information, and registry data) may be used for many types of non-restricted research, including biological and genetic research related and unrelated to the reason for participation in study	Rarely
BCH data use (secondary use, control sample across population)	Identified data can be shared with collaborators on IRB protocol and others at BCH	Rarely
Broad data use	Language of consent allows engagement with other academic networks and industry sponsors to accelerate discovery and therapeutics development	Sometimes

Use of the consenting principles in Table 1 prior to the CRDC was evaluated on 26 research protocols, 10 of which have now incorporated the CRDC consenting principles, 8 of which are in the process of incorporating the principles, and 8 for which incorporation was not preferred or impossible. Very rarely incorporated principles were present in <20% of consent forms, rarely incorporated principles were present in <40% of consent forms, and sometimes incorporated principles were present in <60% of consent forms.

### Recruitment, sample collection, and sequencing

**Fig. 1:**
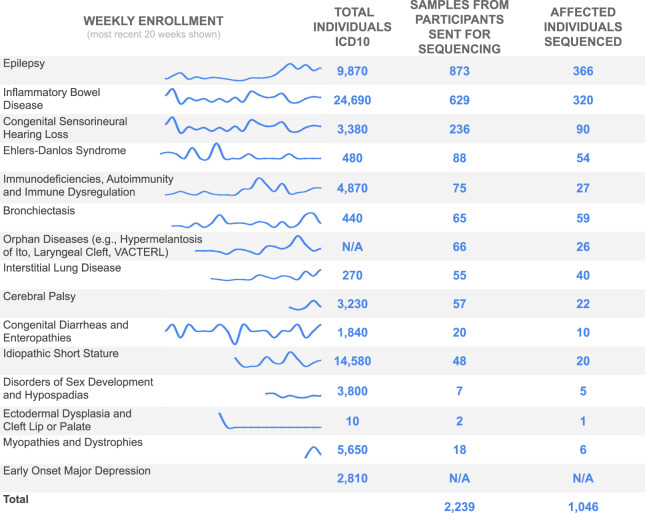
Sample collection. Samples from patients enrolled in disease cohorts. The graphs contain weekly enrollment counts, normalized to average enrollment over the duration of their inclusion in the CRDC; the total number of pediatric patients that have been seen at BCH in the last year with the same ICD10 code; the number of individuals whose samples were submitted for sequencing with the CRDC at GeneDx; and the number of sequenced participants who were affected by the cohort disease.

In order to facilitate high volumes of participant enrollment, we developed a workflow to scale up consenting that relies on research team expertise in identifying research participants. All enrolled patients have been diagnosed with a rare disease that one of the research teams are studying. Each research team identifies patients that match their recruitment criteria by completing reviews of internal patient registries, further supplemented by analysis of medical records. The research teams then contact patients and their families. Internal estimates of patient counts were cross-validated by analysis of BCH patient population in TriNetX^56^, which is utilized as a research database at BCH (Fig. 1). Priority for enrollment within each cohort is at the discretion of the primary investigator (PI) and is governed by each IRB protocol. Factors that are commonly considered include severity of disease, age of patient, lack of informative genetic testing having been previously performed and extreme and/or syndromal phenotypes. Patients are recruited from outpatient clinics and inpatient settings and offered either buccal swabs or blood draws, as governed by each IRB protocol.

Interested participants were initially consented using our standard paper forms, but during the later stage of phase I, research teams were also offered the option to consent using electronic consenting on tablets. On-site consenting was further supplemented by a remote process, which was uniquely supportive of the context and constraints experienced by both study participants and research teams (for example, if a patient did not have an upcoming appointment at BCH or lived far away). In these cases, research teams consented participants over the phone, utilizing mail, email or fax to send blank and signed consent forms.

**Fig. 2:**
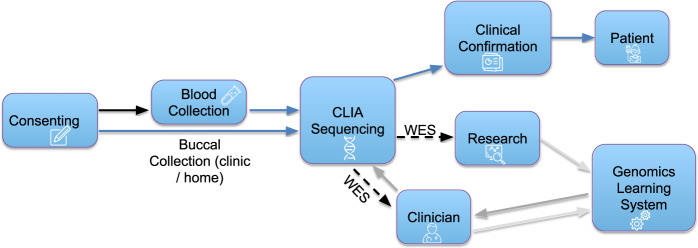
Research to clinical workflow. Patients with or without previous clinical testing were consented to harmonized research protocols. Patients were offered standardized sample collection mechanisms and most patients were dual consented to the Precision Link Biobank to support the collection of additional leftover clinical samples. Patient samples were CLIA sequenced by our sequencing provider (GeneDx) and data was returned to AWS where it was loaded into CRDC infrastructure for analysis. Once research teams identified a candidate variant, analysts worked with clinicians to order the clinical confirmation from the sequencing provider. Clinical confirmations were returned to BCH, added to the patient’s medical record, and communicated to the patient.

In a similar fashion to the CRDC recruitment process, the CRDC sample collection aligned IRB protocols and sample labeling systems. Sample collection is supported by BCH research teams (Fig. 2). Samples are sent to GeneDx (Gaithersburg, MD), a CLIA-certified testing facility, where DNA is extracted and sequenced. All samples undergo CLIA-compliant WES, generating raw data (in the form of FASTQ files), and remaining DNA extracted from each sample is stored in a CLIA-compliant manner. This ensures that excess DNA can be used for future clinical confirmation (i.e., Sanger confirmation and clinical interpretation of clinically relevant variants) or reflex testing without requiring an additional sample (Supplementary Table 1).

### Phenotyping and phenotype data processing

Since analysis of genomic data benefits from access to phenotypic information, we phenotyped participants using automated and manual processes drawing from EHR and research data. Project-specific registries were developed in REDCap^57^, which BCH uses widely as a research Electronic Data Capture (EDC) system. These registries were used to track phenotypic data derived from four sources: (1) manually from research notes (information directly added by the study team); (2) manually from clinical notes or unstructured EHR files; (3) self-reported by the participant to research staff, including via questionnaires; and (4) pulled automatically from the EHR (Supplementary Tables 3 and 4). We harmonized information collected from different research teams’ registries in a central EDC and assigned unique de-identifiers to each participant. Collected phenotypes were mapped to Human Phenotype Ontology (HPO)^58^ terms and stored in the central EDC. Since each research team organized and collected phenotypic data differently, we developed software to integrate data from each research team’s registry and share it to the GLS, as well as GeneDx. The phenotypic information collected by the research teams was very extensive. For example, the IBD team collected project-specific, detailed health information regarding baseline and follow-up visits in their registry. These data included family history, different physical presentations of IBD, classification scores including Pediatric Ulcerative Colitis Activity Index (PUCAI) severity scores^59,60^, dates of specific symptoms and medical interventions, information on medications and patients’ reactions, structured fields automatically sourced from the EHR, and information on past surgeries and hospitalizations.

### Integrative analyses in a GLS

**Fig. 3:**
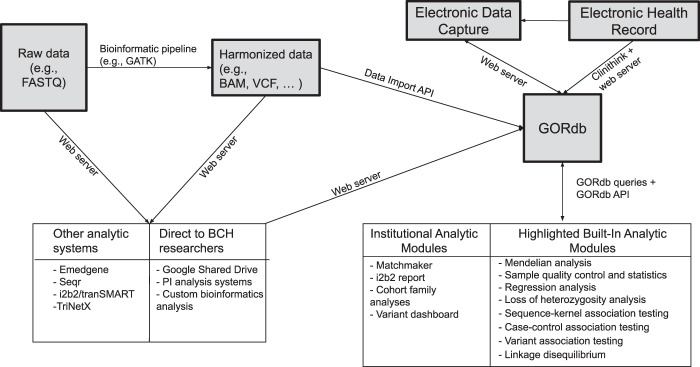
Data flow diagram of genomics learning system. Raw data is processed by secondary pipelines into harmonized data which is ingested into GORdb by the data import API. Phenotypic data from the EDC and EHR are also incorporated. Built-in GORdb queries, as well as institutionally-developed queries operate on the merged data, and can be executed by calling GORdb API or through the WuXi NextCODE user interface. Raw and harmonized data are also made available to other analytic systems and BCH researchers. Information from these systems are fed back into GORdb. Aspects of the GLS are connected by a Python web server, which executes data transfer to/from the GLS components, sends automated alerts to researchers about new data availability and warnings to bioinformaticians about potential metadata errors (for instance, duplicate subject enrollment).

To facilitate analysis, we developed a GLS (Fig. 3) that integrates phenotypic and genotypic data in WuXi NextCODE’s Genomically Ordered Relational database (GORdb)^61^ to support rapid analysis of large cohorts. GORdb’s architecture, which has been previously applied to population scale projects in Iceland^62,63^ and Ireland^64^, readily extended to our current collection. Phenotypic information was derived from the EDC through a validated extract, transform, load (ETL) process (Supplementary Tables 3 and 4) and migrated to GORdb. In addition, de-identified structured EHR data was loaded in Informatics for Integrating Biology and the Bedside (i2b2) star schema^65,66^, which includes diagnoses, medicines, procedures, labs, allergies, vitals, demographics, and specimens (Supplementary Table 5), as well as partially redacted dates for each event. Separately, data from 462 types of unstructured clinical notes were processed by Clinithink’s CLiX Focus software^67^, which uses natural language processing (NLP) to extract HPO terms (Supplementary Tables 6 and 7). As expected, the NLP system was more efficient than manual annotation. Based on a subset of 775 patients with both CLiX Focus and manually annotated HPO terms, we see more HPO terms from CLiX Focus than as were annotated manually. Raw data from the sequencing provider were processed by the bioinformatics pipeline into BAM, GVCF, VCF, and gzipped genomically ordered relational (GORZ) files. As a next step, the processed files were loaded via data import application programming interface (API) into GORdb, where the genomic data was made available alongside phenotypic data on a per-participant basis.

As depicted in Fig. 3, various systems at BCH are integrated with the GLS by connecting to raw data files, harmonized data files, or through direct access to GORdb. WuXi NextCODE also manages the back-end infrastructure in Amazon Web Services (AWS) for GORdb. Most researchers used graphical user interfaces (GUIs) to interact with GORdb, making use of modules that perform different analytic functions. For example, one frequently used module performs analysis of rare variants in affected individuals by integrating genomic information, reference data resources, and inheritance calculations. Other built-in modules enable association tests, regression analysis and linkage disequilibrium calculations. We have extended these built-in modules by developing custom modules using GORdb queries to include cohort family analyses that pool inheritance information, reference data and genomic data across many families to enable researchers to ask questions about genetic carriers and inheritance patterns within a cohort of interest. GORdb is powered by GOR, a full-featured interpreted programming language which combines SQL-like genomic queries with shell scripting to create a diversity of genomics tools. While GOR is designed for genomic investigations, the language has a rich standard library, and provides general tools for mathematics, statistics, and large operations on data tables. GOR programs can be easily packaged into modules and shared with other users, or even other organizations. Users can also select participants matching phenotypic criteria for downstream analysis, leveraging the EHR and other phenotypic information loaded into GORdb, or use a Matchmaker Exchange^11^—like mechanism to seed collaborations. In order to remove nonpathogenic, cohort-specific common variants and optimize analyses^68^, we have also been able to leverage the cohorts together to curate a variant exclusion blacklist that could be applied across the cohorts to filter variants prior to research analysis.

The GLS incorporates standard processes for data governance, including auditing of actions and API access. Other systems that are integrated with the GLS access harmonized data files directly from AWS and return data to GORdb. One system in this category is Emedgene^69^, a machine learning and automated variant classification application used by researchers to prioritize variants in family analyses. Emedgene generates a shortlist of potential causative variants along with supporting evidence, using an automated machine learning engine and a knowledge graph containing gene-disease relationships (Supplementary Fig. 1) and polymorphism variant information. Emedgene’s knowledge graphs are created on a regular basis through the application of NLP to a variety of data sources, including 21,000,000 research articles and hundreds of public databases and proprietary data. This knowledge base currently generates 254,000,000 public domain variants, 179,000 known pathogenic variants, and 26,000 gene-disease connections and is being used to stimulate analyses from all cohorts. Additional systems are similarly connected that provide interfaces to facilitate analysis by users with different needs, including i2b2/tranSMART^70^, TriNetX and Seqr^71^.

The GLS provides access to three distinct types of data: (1) raw data in the precise format that was received from the sequencing provider, (2) harmonized data files that have been processed with a standard bioinformatics pipeline, and (3) integrated data stored in GORdb which includes genomic data, registry data from an EDC, and EHR data that incorporates clinical notes processed by CLiX Focus (Fig. 3). Systems can interface with GORdb by performing standard GORdb queries. Aside from supporting the CRDC projects, implementation of GORdb has allowed us to create controlled, audited instances for datasets that are not broadly consented, including clinical data returned to the hospital and collections from non-CRDC genomic studies being analyzed in GORdb. This accounted for an additional 6075 exomes and 112 genomes being added to the GLS, expanding the entire system to 8516 exomes and 112 genomes. The GLS has also been configured in a way that it is able to present unified data for other advanced analyses by specialized research and bioinformatics teams internally and potentially externally; it also provides a robust platform that can be extended to support external consortia and integrate with additional technologies (e.g., the Genomics Research and Innovation Network^16^, i2b2/TranSMART, TriNetX).

### Genomic findings

**Fig. 4:**
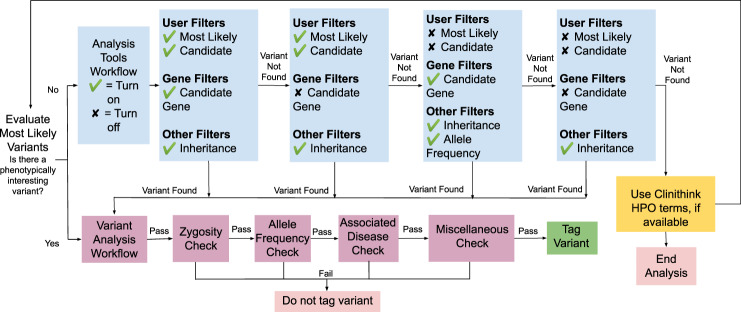
RC variant annotation workflow using Emedgene. Flowchart workflow for evaluating variants prioritized by Emedgene using manually curated HPO terms, as well as CLiX Focus-derived HPO terms.

In order to fully utilize the GLS for gene discovery, the CRDC relied on the disease-specific analysis expertise of each research team. Each team had different internal procedures to identify variants of interest for interdisciplinary review. To further supplement their expertise, bioinformaticians engaged individual study teams to support CRDC research and GLS development, and developed educational courses. Collaboratively with analysts in each of the research teams, the bioinformaticians developed in-house workflows leveraging Emedgene along with manually-curated and CLiX-derived HPO terms to automate, extend and accelerate family-based variant identification (Fig. 4, Table 2 and Supplementary Table 8). They also collaboratively developed in-house workflows leveraging WuXi NextCODE together with phenotypic data from research REDCap databases, CLiX Focus HPO terms, and structured data from the EHR to identify candidate genes between cohorts. In an approach to gene discovery similar to the model developed by the Broad Center for Mendelian Genomics (CMG)^72^, bioinformaticians and analysts within each research team reviewed each case in parallel. These workflows culminated in automated alerts to research team analysts about variants that were considered to be of high importance or of potential interest. Moreover, we have also utilized this team approach to identify potentially clinically relevant variants. In these cases, variants of interest were clinically confirmed using stored DNA extracted from the original sample. Clinical confirmations were returned to BCH, added to the patient’s medical record, and communicated to the patient. In addition, clinically confirmed variants were deposited into ClinVar by GeneDx.

**Table 2:** Identification of variants by different methods.

	Emedgene		WuXi NextCODE	
	Variants analyzed	Average days between upload and variant identification	Variants analyzed	Average days between upload and variant identification
RC analysts	239	26	N/A	N/A
Research team analysts	13	73	36	87

The first batches of data in the GLS have recently been returned to researchers for further evaluation. Though this analysis will continue to expand, we have identified 253 variants of interest in 168 genes leveraging the variant annotation workflow described in Fig. 4 and GORdb modules that identify reported pathogenic and likely pathogenic variants in the most recent secondary findings recommendations from the American College of Medical Genomics (ACMG)^73^. The workflow annotated one or multiple variants for review in about a quarter of families evaluated. Of these variants, preliminary review indicates that one in five (*n* = 57) were known disease-causing variants, two thirds (*n* = 171) were new variants in genes known to cause disease, and the remaining variants (*n* = 25) were phenotype expansions. Further, two in five had either a known drug/disease/gene interaction or an existing relevant clinical trial. The Emedgene-automated ACMG classification scored 20% of the variants as pathogenic or likely pathogenic (P/LP), and the remaining as variants of uncertain significance (VUS).

Research teams’ analyses have led to the clinical confirmation of 43 variants in 32 patients that are new genetic diagnoses and confirmed etiological Mendelian associations (Supplementary Tables 8 and 9) in 29 genes. Some of these patients had atypical or mild presentations and would not typically receive clinical sequencing as a diagnostic for their disease. The research teams that had the highest percentage of their analyzed cases yielding clinical confirmations were those that had the most pre-existing expertise in handling these kinds of data, though research teams with limited prior experience with these kinds of analyses were able to provide meaningful genomic information to their patients through the clinical confirmation of variants. We will continue working with those teams and utilize general reporting to accelerate the clinical confirmation of variants. Reaffirmingly, most (86%) of the clinical confirmations yielded the same ACMG classification as was provided by Emedgene, validating our approach to engage with Emedgene and demonstrating that automation of the research pipeline can streamline and accelerate clinical care. Most importantly, in addition to clinical confirmation we observe many other activities accelerated by the GLS, including greater evaluation of genomic data on a per-cohort and cross-cohort basis. Other variants are in various stages of review by research team analysts, interdisciplinary research teams, or have been submitted to Matchmaker Exchange^11^, and a handful of new genotype-phenotype associations and many phenotype expansions have been preliminarily identified. Further, new collaborations and functional studies have been initiated. Beyond providing clinical confirmations to families, research teams also connected families with other services. For example, one family with two affected children were connected to a clinical trial at a nearby hospital for which they were eligible based on their genetic diagnosis and another could enable a patient with an atypical presentation to be seen at a specialized multidisciplinary clinic.

## Discussion

In this report, we outlined how a genomic medicine initiative forged consensus across the institution, how rapid collection of uniform data accelerated the refinement of a comprehensive analysis platform, and how these processes and technologies lend themselves to accelerated utilization of genomic data in support of research and patient treatment. As the strategic initiative finished its first phase, we streamlined the consent model, sample collection process, and technologies used at BCH, lowered the barrier to entry for new investigators conducting genomics research, and reduced the bioinformatics burden for researchers with ongoing genomics research. We created a database of broadly available genomic, research, and clinical data, improving access to genomic data for clinicians and researchers; to our knowledge, this is the most comprehensive database of genomics and phenotypic data focused on pediatric rare disease patients. We deployed collaborative analytic tools, merged genomic data into preparatory-to-research queries, and streamlined research to clinical pipelines enabling precision medicine results faster and with fewer blood draws. The CRDC establishes a technical and administrative roadmap for hospitals establishing integrated genomics learning systems; the future of rare disease precision medicine research will hinge upon national and international collaborative systems.

Though researchers have been conducting genetic and genomic research at BCH for three decades, the establishment of the GLS by the CRDC represents the first implementation of harmonized and shared genomic, research and clinical data at scale. The GLS we have developed will continue to be extended and improved to meet the needs of researchers and clinicians. Each of our decisions around consenting, sequencing, and analysis has shaped downstream processes and introduced opportunities and challenges which we will discuss here.

One of the biggest opportunities that was underscored by the project was the ability to effectively merge consenting across the institution and bring them to a unified framework. Our approach of modifying research teams’ protocols with the CRDC genomic framework was fairly quick and compatible with dual enrollment into Biobank. In contrast, the use of a centralized consenting approach, such as the institution-wide Precision Link Biobank or the Manton Center protocols, was not positioned to swiftly secure genomic research consents across all of the supported studies. The main constraints pertained to return of results (not part of the Precision Link protocol) and complexities in consenting provenance. Additional benefits of the current multi-protocol approach include reliance on the participating study teams for enrollment, sample collection, education, and follow-up and that it allowed us to bootstrap the project without establishing a separate core. This current model will continue to be evaluated as the project continues, and we recognize that a centralized approach, such as the institution-wide Precision Link Biobank protocol^10^, could potentially lead to additional efficiencies and might further improve the quality of clinical follow-up. A centralized consenting approach would also have the added benefit of standardizing how we address secondary clinical findings where the initiating study team is lacking expertise and capacity for those findings.

Not surprisingly, given close interactions between BCH clinicians, researchers, and patients, the rates of enrollment were high in phase I. We believe that the use of buccal swabs and remote consenting helped greatly with enrolling more participants and sequencing more complete families. Even when patients visited a BCH clinic, many families had at least one family member who was remotely consented, as frequently only one parent attended the hospital visit. In order to further support the remote consenting process we introduced an electronic remote consenting system, which is similar to the electronic consenting system used at BCH locations and also utilizes REDCap. Notably, though most of the research teams still prefer in-person paper consenting to electronic consenting on tablets, they are enthusiastic about electronic remote consenting options. We will continue to grow these cohorts in phase II of the initiative; in cases where the BCH population is not significant, we may extend them with data from other pediatric institutions.

The narrowing of the gap in pricing between CLIA-compliant and research sequencing creates unique opportunities for research hospitals. Here, we opted to sequence samples under CLIA guidelines. The Center for Medicare and Medicaid Services (CMS) regulates that all laboratory testing (except research) performed on humans in the United States occur in a CLIA-compliant manner^74^. CLIA sequencing laboratories are responsible for rigorous sample tracking processes and quality controls, therefore sequencing services provided under CLIA have been more expensive and more rarely used in research. Benefits of following CLIA workflows include cost avoidance of additional sample extraction, as well as added speed and simplicity when performing follow-up clinical testing. Moreover, research sequencing benefits when captured in a CLIA-compliant manner from improved pre-analytic handling of samples and quality control^75^. As the gap between CLIA and research sequencing costs is closing, the additional costs of CLIA sequencing have been endorsed by BCH clinicians and others^13,76^. We expect that CLIA sequencing providers will close the gap to adjust to increased research demand. More broadly, the distinction between research and clinical genomics is blurring; research findings are being integrated into clinical application when analyzed in the appropriate environment and data from clinical sequencing is being used for research. Increasingly, clinicians at BCH are reviewing clinical genomic data through the GLS. As these data are readily shared to researchers when consent is obtained, the GLS forms a basis for combined clinical-research workflows. With access to the GLS, clinicians may leverage shared modules for analysis, existing research data, and methodologies in their evaluations. Utilizing CLIA sequencing processes upfront enables rapid and seamless return of findings from researchers. We expect that researchers and institutions will find increasing relevance of CLIA processes, with potential long-term benefits including avoidance of additional sample collection and testing and suitability to creating merged genotypic, phenotypic and clinically sourced databases.

WES is the most frequently used NGS modality in clinical application and is particularly effective at identifying single nucleotide variants and small indels in coding exons, as well as flanking intronic sequences involved in normal splicing^77^. Of the 253 variants of interest we have thus far identified, 14 were outside of exons. Access to the entire genome would enable the broad identification of variants in non-coding regions, as well as improved identification of copy-number variants (CNVs)^78,79^. A shift to WGS would necessitate the collection of whole blood to increase sensitivity, particularly when calling CNVs^80^. Utilizing blood collection would also enable the analysis of other biological molecules (e.g., RNA-seq), which currently would only occur through the collection of a second research sample at the time of enrollment or via patient re-contact. CLIA WES costs less than research WGS, allowing us to maintain a rigorous CLIA process at scale. Moreover, data storage and transit costs are lower as the average size of a WES BAM file is many times smaller than that of a typical WGS BAM. These data management considerations also translate to compute costs as BAM files are processed to VCFs and integrated into GORdb. Most importantly, the approach to utilize buccal swabs in support of WES allowed us to dramatically scale up rates of sample collection, particularly in pediatric patients unable to donate blood and second parents that are not typically present at the hospital at the time of enrollment. In the CRDC’s second phase, we plan to pilot transitioning CRDC sequencing to WGS.

The development of the GLS as a centralized institutional resource has enabled research teams to offload maintenance and information technology work, allowing them to focus on research. Research teams confirmed that the GLS functionality, interfaces, training and support met their needs. While other integrated genomic data systems are available, such as Hail^81^ and Genome Query Language,^82^ GORdb benefits from integration of back-end infrastructure, APIs, modules and GUI for the platform. This is integral for the project because WuXi NextCODE’s GORdb robustly handles large repositories with many thousands of samples, while remaining accessible to clinicians, researchers, and liberating bioinformaticians from the task of managing compute clusters directly. Because of the extensible nature of the system, additional modalities, such as methylation, immunoprecipitation, chromatin capture and microbiome analyses, can be readily incorporated and integrated together. Extending the other facets of the GLS to these additional modalities would leverage similar workflows as the existing WES/WGS infrastructure: raw data is processed into harmonized data that is uploaded to GORdb and other systems. These extensions would require deploying additional algorithms to the bioinformatics pipeline, and development, testing, validations and deployments of new GORdb queries, which would be used to integrate additional data with existing merged information in GORdb. WuXi NextCODE has already developed built-in modules for the analysis of CNVs and RNA-seq data. We have started using the CNV modules and incorporated 19 CNV profiles in the GLS. Further, the built-in GORdb API enables analyses that leverage the compendium of common bioinformatics tools, such as those available through Bioconductor^83^ or Bioconda^84^. Users can create and share custom modules within GORdb and less experienced researchers can leverage existing modules. Since de-identified genomic, research and clinical data is merged within GORdb and shared across BCH, the merged GORdb data lends itself to analyses aggregated across cohorts, such as reviewing if any patients in different cohorts cluster together or producing adequate statistical power for association studies. Unified analyses of standardized homogeneously collected genomic and phenotypic data from large cohorts of different rare or Mendelian diseases has been shown to be a powerful approach for genetic discovery^13,85^.

Carefully reviewed use of commercial vendors generally accelerates the use of cutting-edge technologies, mitigates costs, and allows research teams to remain focused around their leading expertise in rare diseases and discovery science. Some of the critical commercial contributions to the CRDC include CLIA-compliant research sequencing by GeneDx, secondary and tertiary analysis of datasets by WuXi NextCODE in an Amazon AWS Cloud environment, Emedgene variant prioritization with machine learning algorithms and AI knowledge graphs, and NLP of clinical notes by CLiX Focus. Systems such as TriNetX and i2b2/TranSMART, developed at Harvard in collaboration with BCH’s Computational Health Informatics Program, and other tools and utilities developed at BCH, extend and interconnect various CRDC platform components and will allow us to support potential data sharing with other institutions. Taken together, we find that a heterogeneous infrastructure combining systems and modules developed in-house with third-parties allowed us to rapidly meet requirements specific to BCH researchers or processes, while simultaneously retaining the ability to scale without needing to fundamentally reconfigure our architecture. The strategy we deployed to develop a GLS that integrated commercial technologies and in-house pipelines enabled us to rapidly deploy new systems while maintaining a lean in-house team of bioinformaticians. Licensing WuXi NextCODE, Emedgene and Clinithink enabled our GLS to have the scalability of GORdb, the AI of Emedgene and the NLP of CLiX Focus, each of which would have required large bioinformatics teams to have developed in-house and maintain, freeing CRDC bioinformaticians to focus on BCH context specific tasks. While challenges around data security and patients’ privacy^86^ persist, we conclude that in order to accelerate the pace of genomic research at pediatric research hospitals, a delicate interplay between academic and commercial entities will continue to be necessary.

While the cost of genomic sequencing continues to decrease, other expenses, including patient recruitment, data management and data analysis, remain largely unchanged. In order to accelerate genomic research, all research hospitals face challenges in identifying potential funding sources, third-party vendors, and commercial partners to advance the use of data for therapeutic development. The hospital decided to support the current project in order to stimulate the use of genomic data in research, the clinic and education. As an outcome, all CRDC datasets remain the property of BCH and third-party vendors can only access data in support of BCH-contracted work, as per the terms of the BCH Business Associate Agreement (BAA). Though the hospital has not yet decided to share the genomic data with other academic or commercial entities, all enrolled patients have consented to such data sharing, with the understanding that this would be reviewed by BCH’s Institutional Genomic Oversight Committee for use in the development of new therapeutics and to further our understanding of human disease. Moreover, legal documentation (e.g., Data Use Agreement, BAA) required to share data would be secured and filed with the IRB. Perhaps most importantly, while the majority of technical and procedural processes have been streamlined, we are only now entering the phase of the project where the collected genomic data can be fully exploited and utilized by an extensive network of research laboratories at BCH, potentially in collaborations with scientists from other academic institutions. These partnerships have the potential to translate emerging findings, in line with recent projects from BCH^3^.

In this report, we describe how we scaled workflows and infrastructure for cohort sequencing at BCH to provide support for genomics research. We describe the data that was collected and how it was integrated into a GLS, combining commercial applications and custom development in a secure and transparent manner. We discussed the value of these data and the GLS to patients, researchers and clinicians. We have provided a model for institutional integration of data to support both discovery and clinical use. This work shows that there is value in aligning consent, developing a modular GLS and integrating clinical and research data sources and that being able to do this depends on the contributions of many stakeholders. We have demonstrated the viability of this system by rapidly enrolling, sequencing and reviewing data from over 2400 participants, and research is ongoing. The first phase of the project established the collection, integration, and sharing of research, clinical and genomic data within the GLS. In the next phase, the focus will shift to data analysis, integration with other institutions and integration of other modalities, including WGS. The approaches and lessons from our work serve as an example for developing institutional workflows for the integrated analysis of research, clinical, and genomic data. Adoption of comparable strategies by other institutions will further enhance research and clinician collaborations, provide greater power for genomic analyses and accelerate pediatric precision medicine.

## Methods

### Study design

This work describes processes and results from the first year of Boston Children’s Hospital’s (BCH) Children’s Rare Disease Cohorts initiative. Phase I of this initiative began on October 1, 2018 and continued through September 30, 2019 at BCH, Boston, MA, USA. Phase II is ongoing and began on October 1, 2019. In Phase I, 2441 participants were enrolled. Genomic findings presented in this work strictly relate to these 2441 CRDC participants. This paper also describes our work in creating a common genomics learning system (GLS) for research and clinical genomics activity at BCH. The GLS contains a wide range of research and clinical genomic data. We integrated 6075 additional, non-CRDC exomes and 112 genomes during the first phase of the CRDC. These patients and family members were enrolled in studies that began prior to the CRDC initiative, and their data are not in general shareable. We do not present any results from analysis of these patients. The BCH IRB approved all research related to this study and written informed consent was obtained from all research participants.

### Sample collection

Samples for the CRDC were collected as blood (*n* = 146) or buccal swabs (*n* = 2093). The remaining participants had previous or upcoming clinical sequencing scheduled (*n* = 72) or did not complete the sample collection process (*n* = 130). Buccal swabs were collected either in clinics or at home. Patients who were consented remotely were typically mailed self-administered buccal swab kits with a return envelope included.

### Sequencing

GeneDx extracted DNA using IDT xGen probes. The average coverage across the WES was 100× and more than 95% of targets were covered at 20×. The GeneDx medical exome (4500 genes) had 99.4% of targets covered at 20× and an average depth of 114×. All sequencing data passed specific minimal quality control requirements, including pass-filtered sequencing yield of 4GB, thresholds for mapping percent to hg19 (>95%), target coverage at 10× (90%, 97–98% typical), mean target coverage (50×, average 100–120×), duplicate read percentage (<30%, <10% typical), and read-quality metrics (80% Q30).

### Phenotyping and phenotype data processing

The de-identifiers were configured for each family and participant based on their relationship to the proband. The vast majority of fields were manually sourced from research notes, followed by manually sourced from clinical notes or the unstructured EHR. The least frequently used source of data was self-reported. For example, race^87^ was derived from a self-reported questionnaire in the IBD cohort, originated from research notes for the epilepsy cohort, and was pulled from the EHR for other cohorts. Fields such as name, date of birth (DOB), or gender were frequently manually curated from clinical notes, but in some cases, they were pulled automatically from the EHR. Others (such as medical record number (MRN), family relationship information, and tracking information, including date consented, date and type of sample collected, sample collected location) originated from research records, many of which were added to REDCap databases during CRDC on-boarding (Supplementary Table 4). The remaining fields that were collected (e.g., ethnicity^87^, disease condition information) were sourced heterogeneously. Like the consenting and sample collection processes, patient phenotyping relied on research team expertise. Though timestamps for each event are stored in the EHR, the collection of temporal phenotype data in the research team’s registry is at the discretion of each research team and depends on disease specific characteristics.

### Bioinformatic pipeline and harmonized data access

Once sequencing was completed, the raw WES data were returned within four weeks via an automated upload of FASTQ files to BCH’s Amazon Web Services (AWS) account and archived in a Simple Storage Service (S3) bucket (Fig. 3). There the WES datasets were uniformly processed through a standard variant calling pipeline managed by WuXi NextCODE^61^ in a research environment. Adapters were trimmed using Skewer v0.2.1^88^, FASTQ analysis was performed by FastQC v0.11.7^89^ and base quality was calculated using BBMap v37.97^90^. Read alignment, read depth calculation, realignment, recalibration, and variant calling were performed by Sentieon v201808.03^91^: BWA, HSMetricsAlgo, WGSMetricsAlgo, markduplication and Realigner, QualCal, Haplotyper and GVCFtyper^91^. Verifybamid 1.1.3^92^ was used to check contamination and GATK 4.1.2.0^93^ to count reads in bins. WuXi NextCODE GORpipe 4.3.0^61^ converted other variant data to genomically ordered relational (GOR) format and annotated variants with VEP 96.2^94^ and custom tools. Processed data (BAM/VCF files) were stored in AWS S3 buckets and are used to support other institutional databases such as i2b2/TranSMART^70^ and the Precision Link Biobank^10^. In the future, with an oversight from BCH and IRB, this access could be extended to other cross-institutional projects, such as Genomics Research and Innovation Network (GRIN)^16^ (Fig. 3). S3 or API access was provided to each system to avoid duplication of data storage.

### Data governance

The GLS contains de-identified data from many studies, and the EDC and EHR components manipulate personally identifiable information (PII). Therefore, there are federal and institutional regulations that control access to this data. Each of the components of our workflow have features for access control that we were able to leverage in the GLS. PII is stored only in the EDC and EHR. Policies around data access in research team REDCap databases are institutional. Each REDCap project is tied to a specific IRB protocol, and access is only permitted to researchers on that protocol. Within a REDCap project, access can be further configured by using REDCap data access groups. Data in GORdb and the GLS analysis systems are all de-identified and built-in facilities are used for user authentication and data siloing. GORdb, Emedgene and Seqr are each able to employ project-based access control and user and administrative actions are logged in all systems. Data consistency across systems is maintained with regularly scheduled (daily or weekly) synchronization from our central REDCap database, managed by the Python web server, which runs a set of automated jobs. This REDcap acts as a source of truth for the entire GLS: data is pushed out from it to the downstream services. Data is fed into the central GLS REDCap from smaller cohort-specific or clinical REDCaps, but this data is validated and filtered before becoming integrated with the GLS.

Harmonized genomic data is made available to systems via Identity and Access Management (IAM) policies in AWS, which control read-only access to the S3 buckets. Harmonized and raw data is also available to research teams via investigator-requested read-only access to cloud storage. Data standardization and quality controls are handled with a number of overlapping methods. Sample and subject metadata is managed through a central REDCap and a Python web server, which records and validates subject consent, sample collection, receipt of raw sequencing, and data harmonization. The web server that handles data transfer from EDC to GORdb performs basic validation and error-checking on raw participant data from the research lab. Further, this server unifies data formatting across all cohorts by enforcing a common data structure for all participant data (Supplementary Table 3). Automated error reporting alerts CRDC bioinformaticians to potential data errors, and validations are performed with other BCH clinical and research datasets from the EHR and the BCH Biobank to find other inconsistencies.

### Clinithink CLiX Focus quality control

CLiX Focus extracts HPO terms on a per note basis and provides a note-frequency ranked list of HPO terms for each patient. The HPO terms at the top of the frequency ranked list are those that CLiX Focus found most often in clinical notes. HPO terms that were infrequently identified in notes are less likely than those identified multiple times to be a phenotype observed by clinicians. Unlike manual processes, CLiX Focus annotates patients with all relevant parent HPO terms in the hierarchy rather than just the most specific terms (e.g., where manual processes would annotate only focal seizures (HP:0007359), CLiX Focus’ CNLP would annotate focal seizures (HP:0007359), seizures (HP:0001250), and abnormality of the nervous system (HP:0000707)). Even though terms higher in the hierarchy are also annotated, more than half of the annotated HPO terms are terminal terms, and do not have a child term in the results.

Extensive validations have previously been conducted to optimize application of the CLiX Focus methodology for pediatric rare disease diagnosis in a separate health care system^41^, including validation regarding the use of hierarchical parents of extracted terms. We tuned the CLiX Focus system to BCH’s EHR construct and tested and validated the results utilizing a cohort of rare disease cases from The Manton Center for Orphan Disease Research. We applied the CLiX Focus NLP engine to individuals for whom detailed HPO annotation had already been conducted manually by trained researchers. For six test cases, the CLiX Focus output was compared with the manually extracted HPO terms and CLiX-derived HPO terms were scored as a true or false positive. We were able to leverage manually annotated HPO terms to determine a comparable recall as has been previously reported for CLiX Focus HPO terms (85%)^41^, but this comparison does not lend itself to meaningful precision or sensitivity calculations. This analysis was the basis for discussions on how to update the CLiX ENRICH rules and what types of notes should be included or excluded from the processing based on commonly identified problems. Some rule changes were necessitated by the specific structure of BCH clinical notes. Common reasons for false positives included imputation of specific eponymous diseases based on the names of patients or their health care providers, assignment of terms based on checklists of findings reported absent following a colon, and diagnoses associated with gene symbols contained in a differential list of diagnoses. Based on this analysis, the selection of input document types was also adjusted to retain intake and discharge notes while excluding in-patient status updates, pre-MRI screening questionnaires, nursing notes with checklists of potential findings, and other document types with repetitive nature and/or low information content (Supplementary Table 6).

Using the above filtering criteria, the average CRDC patient had 510.8 clinical notes in their medical records and CLiX Focus extracted 191.9 HPO terms on average, including parent terms. On average the top HPO term found in patient notes was found in 44.6% of notes. For example, a patient enrolled in the epilepsy cohort with 176 processed clinical notes was observed to have 50% (*n* = 88) of their notes containing terms that mapped to seizures (HP:0001250). Before being loaded to GORdb, an additional frequency-based filter was leveraged to take advantage of the high number of notes per patient at BCH and to further enrich for phenotypes that had been observed by multiple clinicians. We applied heuristic filters to the HPO terms extracted by CLiX Focus and applied different filter cutoffs for patients with low (<20), moderate (20–50), or high (>50) HPO terms extracted (Supplementary Table 7), yielding an average of 45.9 HPO terms per patient.

### Reporting summary

Further information on experimental design is available in the Nature Research Reporting Summary linked to this article.

## Supplementary information

Supplementary Information

Reporting Summary

## Data Availability

Data sharing is available internally to investigators at BCH through the GLS and may be available upon request for collaborative projects between pediatric or other institutions. 32 exomes have been made available in the European Genome Archive, accession ID EGAD00001006179.

## Code availability

Code may be available upon request.
