# Supplementary material for: Children’s rare disease cohorts: an integrative research and clinical genomics initiative
Source: NPJ Genom Med. 2020 Jul 6;5:29. doi: 10.1038/s41525-020-0137-0 (PMC7338382; doi:10.1038/s41525-020-0137-0)
Supplement: Supplementary file 1 — Supplementary Information [file 41525_2020_137_MOESM1_ESM.pdf]

Supplementary Figures

Supplementary Figure 1 | Representation of Emedgene's Knowledge Graph for a pair of compound heterozygous variants in a congenital diarrhea patient.

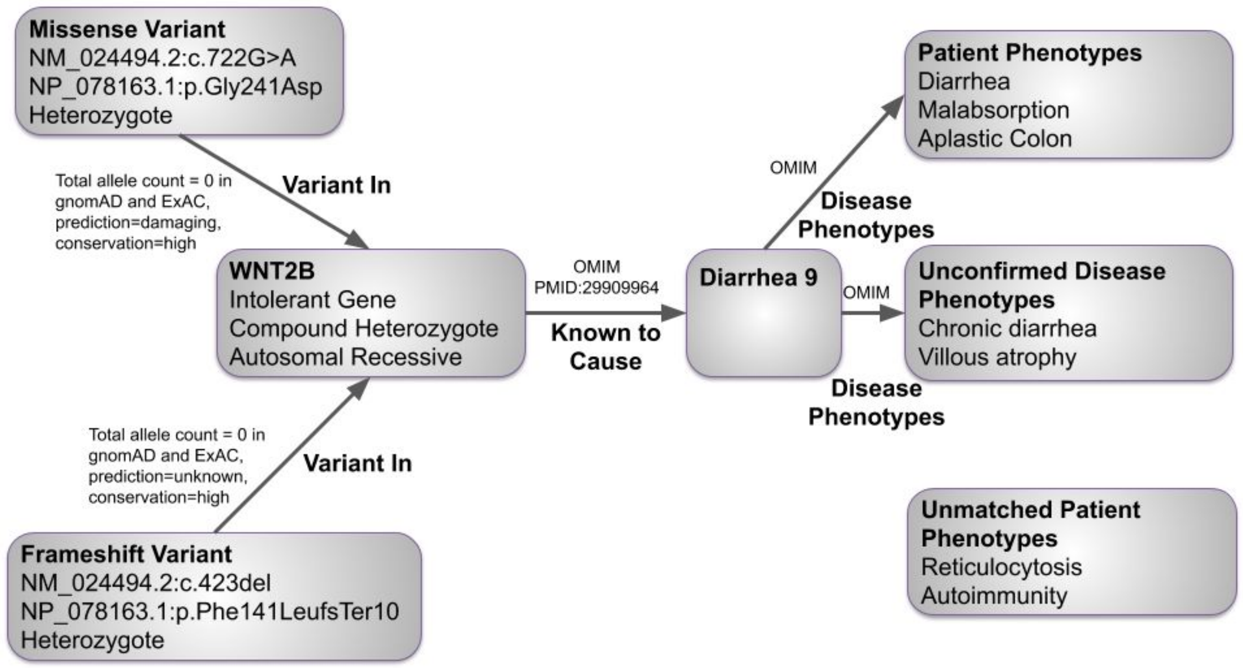

Example HPO terms are shown in the patient phenotypes, unconfirmed disease phenotypes and the unmatched patient phenotypes boxes that were derived via clinical NLP by CLiX Focus.

Supplementary Tables

Supplementary Table 1 | Consenting Principles

<table>
<tr><th>Consenting Principles</th><th>Reasoning</th></tr>
<tr><td colspan="2">Rights and Interests of the Patient</td></tr>
<tr><td>Identified variants are clinically confirmed</td><td rowspan="3">Consent modifications in research protocols enabled participants to delineate between receiving primary, primary and secondary, or no findings, gave them the option to have research findings clinically confirmed, and the ability to learn about additional research studies or clinical trials that might benefit them.</td></tr>
<tr><td>Participants opt for return of primary results, primary and secondary/incidental, or neither</td></tr>
<tr><td>Participants consent to re-contact to request additional data/samples and interest regarding being offered enrollment in other research studies</td></tr>
<tr><td colspan="2">Sample and Data Flow</td></tr>
<tr><td>Research consents contain language regarding the use of previously collected clinical data</td><td rowspan="4">Other consenting principles, including operational language that enabled researchers to leverage hospital infrastructure for sample collection, storage and consenting were applied.</td></tr>
<tr><td>Remote consenting and e-consenting are available</td></tr>
<tr><td>Support Biobanking at the BCH Biobank</td></tr>
<tr><td>Consent allows the identification of genetic factors</td></tr>
<tr><td>Consent enables identified CLIA sequencing upfront for streamlined confirmation</td><td>A key aspect of the CRDC workflow was sending identified CLIA compliant samples for sequencing. Clinical-grade research sequencing enabled the streamlining of research findings to the clinical environment and for researchers to work with clinicians to order clinical confirmations without re-consenting the patient or obtaining a new clinical sample. To support this workflow, novel operational language was added to each informed consent document that allowed investigators to send identified samples for CLIA sequencing.</td></tr>
<tr><td colspan="2">BCH Data Use</td></tr>
<tr><td>Samples and data (genomic sequences, medical record information, and registry data) may be used for many types of non-restricted research, including biological and genetic research related and unrelated to the reason for participation in study</td><td rowspan="2">Broad use and data sharing language in the informed consent enabled data and samples to be used for many types of unrelated research, for identified data to be shared within BCH, and for de-identified data to be shared with consortia, external repositories and industry. Together, the incorporated language enabled researchers to leverage hospital infrastructure for sample collection, data distribution and consenting, which further streamlined these processes.</td></tr>
<tr><td>Identified data can be shared with collaborators on IRB protocol and others at BCH</td></tr>
<tr><td colspan="2">Broad Data Use</td></tr>
<tr><td>Language of consent allows engagement with other academic networks and industry sponsors to accelerate discovery and therapeutics development</td><td>De-identified data can be shared with consortia, repositories, and industry to accelerate discovery and therapeutics development</td></tr>
</table>

Supplementary Table 2 | IRB Modification Days to Draft Submission and Received Amendment Approval

	Existing Protocol		New Protocol	
	Days to Draft Submission	Days to Approve Amendment	Days to Draft Submission	Days to Approve
Fall 2018 cohorts (n=2), all approved	71	51	N/A	N/A
Spring 2019 cohorts (n=8), all approved	54	42	72	100
Summer 2019 cohorts (n=5), 3 approved	0 (already modified, n=2)	0 (already modified, n=2)	55	24

Average number of days research teams with existing or new protocols took to draft their IRB submission. In the case of existing protocols, this would be an amendment to an existing IRB protocol. In the case of new protocols, this would be to draft a new IRB protocol. Included also are the number of days taken to approve the IRB protocol or amendment by the IRB, including clarifications in response to comments from the IRB.

Supplementary Table 3 | Metadata

Field	Options	Example research team	How was the data harmonized from the original REDCap database?	What was the data source for this field?
Standardized Identifier	Generated	All research teams	BCH-YY-NNNNN-XX*	1
Name	Free text	IBD	Unchanged	2
		Epilepsy	Unchanged	2
		Example	Parsed	4
DOB	Date	IBD	Unchanged	2
		Epilepsy	Unchanged	2
		EDS	Unchanged	4
MRN	Free text	IBD	Unchanged	1
		Epilepsy	Unchanged	1
		Example	Unchanged	1
Date consented	Date	IBD	Unchanged	1
		Epilepsy	Unchanged	1
		Example	Unchanged	1
Race	Multiple choice: per NOT-OD-15-089	IBD	Parsed	3
		Epilepsy	Unchanged	1
		Example	Unchanged	4
Ethnicity	Multiple choice: per NOT-OD-15-089	IBD	Unchanged	4
		Epilepsy	Unchanged	1
		Example	Unchanged	4
Gender	Multiple choice: Male, Female, Unknown	IBD	Unchanged	2
		Epilepsy	Unchanged	2
		Example	Unchanged	4
Relationship	Multiple choice: Index, Mother, Father, Sibling, Monozygotic Twin, Dizygotic Twin, Case, Control	IBD	Parsed	1
		Epilepsy	Unchanged	1
		Example	Unchanged	1
Affected status	Yes / No	IBD	Parsed	1
		Epilepsy	Unchanged	1
		Example	Parsed	1
Family ID	Free text	IBD	Parsed	1
		Epilepsy	Unchanged	1
		Example	Added	1
Type of Sample Collected	Blood, Buccal Swab, Clinical	IBD	Parsed	1
		Epilepsy	Parsed	1
		Example	Added	1

Date sample collected	Date	IBD	Unchanged	1
		Epilepsy	Unchanged	1
		Example	Unchanged	1
Location blood drawn	Experimental Therapeutics Unit, Phlebotomy	IBD	Parsed	1
		Epilepsy	Added	1
		Example	Added	1
Location buccal swab collected	Multiple choice: BCH, At home	IBD	Added	1
		Epilepsy	Added	1
		Example	Added	1
HPO terms***	A list of HPO terms: [HP00001,HP001234...], built differently for each group.****	IBD	Parsed	1, 2 and 4
		Epilepsy	Added	1, 2 and 4
		Example	Variable	1, 2 and 4
Examples of lab-specific data collected***	Various formatting, usually stored as free text or numeric data in the genomic cohort database****	IBD	Unchanged	2
		Epilepsy	Unchanged	1
		Example	Unchanged	2

Whether the data was parsed, added or unchanged was variable across the cohorts. Included is the data source, (1) research notes (unique information directly added by the study team); (2) self-reported by the patient to research staff, including via questionnaires; (3) the structured EHR; and (4) clinical notes or unstructured EHR files *YY corresponds to the year the patient was first consented, NNNNN is a randomly generated number for the family, and XX is a number corresponding to who the person is within the family. **Some use REDCap's HPO validation service to enter HPO terms directly (unchanged). Others have phenotype-specific questions, and responses to these are matched to distinct HPO terms (parsed and at times partially added). ***HPO terms and lab-specific data collected may have time-dependent information. Timestamps are collected at the discretion of individual research groups and are not currently standardized. **** includes PUCAI scores for IBD.

Supplementary Table 4 | Phenotype Collection Prior Use.

Areas	Phenotype Collection	Use in Lab REDCap Projects Prior to CRDC On-boarding
Patient PHI	MRN	Existing
	Name and Date of Birth	
Genomic Metadata	Basic Information: Gender, Ethnicity, Race	Existing
	Family Information: Family ID and Relationship	Variable
Phenotypic Metadata	Research collected HPO terms	Variable
	CLiX Focus HPO terms	N/A, parsed from clinical notes
	Affected Status	Variable
Sample and Data Flow	Date Consented and Date Sample Collected	Variable
	Type of Sample	Added
	Location of Blood Draw or Buccal Swab	

While the details varied between labs, there were common patterns to the REDCap schema changes needed to onboard labs to the Cohort Sequencing project: (1) Almost every lab had been collecting patient PHI and ethnicity information prior to onboarding; (2) labs had typically been collecting family ID and relationship information if they were doing genetic testing or large-scale sample collection (biobank samples from family members), other labs were proband-only and needed modifications to identify family members; (3) every lab had been collecting phenotypic data, however, only some had collected this data formatted as HPO terms; (4) the proband-only labs did not directly mark affected status since every proband was affected, and needed REDCap changes to mark that family members may be unaffected; (5) the type of sample and location of blood / buccal collection are specific to the Cohort Sequencing process and had to be added to almost all REDCap databases. Even labs that had been collecting samples previously needed an update to annotate where the samples were collected and that the samples were being sent to GeneDx for sequencing.

Supplementary Table 5 | Data was transformed from an observation-centered schema to a patient-centered schema.

**Patient-focused relational data**	**Fields pulled**	**Public health ontologies the concept code maps to**
Diagnosis	ICD code, ICD description	ICD9 and ICD10 diagnosis codes
Medicines	internal code, description (separate fields for medicines administered versus prescribed)	N/A
Procedures	ICD9/ICD10 procedure or CPT code, ICD9/ICD10 procedure or CPT description	ICD9 Proc, ICD10 PROC and CPT4 codes
Vitals	internal code, descriptions of the vitals taken, the numeric value of the specific vital	N/A
Specimens	the code for a type of specimen in the BCH Biobank, the text description of a type of specimen, description of container, temperature of specimen	N/A - internal schema for BCH Biobank
Allergies	Internal code, description of allergy and severity	N/A
Demographics	Internal codes and descriptions for sex, language, race, name, marital status	N/A

Data is stored in the star schema by row, where rows can include many different kinds of observations (eg., medicines, lab values, vital signs), during the transformation into a patient-centered schema, different kinds of observations were grouped. Major groups include: diagnosis, medicines, procedures, labs, allergies, vitals, demographics. International Classification of Diseases (ICD), 9th and 10th Revision codes (ICD9 and ICD10); Current Procedural Terminology (CPT), 4th Edition; ICD9 Procedure (ICD9 Proc); ICD10 Procedure (ICD10 Proc).

Supplementary Table 6 | Types of notes included in CLiX Focus.

Note Type	Number of Note Subcategories	Examples
Clinic Note	196	Genetic Counseling Clinic Note, Neurosurgery Clinic Note
Consult	69	Endocrinology Consultation, Palliative Care Consultation
Admission	21	Cardiology Admission, Pediatrics Admission
Visit	29	Nephrology Visit, Pulmonary Visit
Inpatient	27	Gastroenterology Inpatient
Other (imaging, etc)	124	MRI Brain, Bone Scan Whole Body, PFT Interpretation

Many (982) note types were not included in the NLP process, ex. Anesthesia Followup.

Supplementary Table 7 | CLiX Focus Filtering Heuristics.

# Of CLiX Focus HPO terms	Average # of Clinical Notes	Filtering Criteria
20 or less (8.6% of patients)	10.5	No further filtering
20 - 50 (3.6% of patients)	29.6	< 5 notes per HPO term
50+ (87.8% of patients)	540.2	Bottom 10% of HPO terms by frequency *

* The HPO term that appeared in the most notes for the individual is used to calculate the 10% cutoff. For example, if the most frequent HPO term occurred 200 times, any term that occurred with a frequency of less than 20 would be filtered out.

Supplementary Table 8 | Identification of Variants by Different Modalities.

	Variants of Interest Identified using Manually Curated HPO terms	Additional Variants of Interest Uniquely Identified using CLiX Focus HPO terms
Variant Analysis Workflow (Figure 4)	170	10
Analysis Tools Workflow (Figure 4)	69	4

Table with information regarding the number of variants found through different modalities. An additional 22 P/LP variants were found via a GORdb module that queries P/LP variants in genes from the most recent secondary findings recommendations from the ACMG.

Supplementary Table 9 | Clinical Confirmations Per Cohort.

Cohort	Number of Clinical Confirmations Ordered
Epilepsy	13
Congenital Sensorineural Hearing Loss	7
Immunodeficiencies, Autoimmunity and Immune Dysregulation	5
Inflammatory Bowel Disease	2
Ehlers-Danlos Syndrome	1
Idiopathic Short Stature	1
Orphan Diseases	2
Congenital Diarrheas and Enteropathies	1
